# The Effect of Ultrasonic Vibration on the 3D Printing Fabrication and Grinding Performance of Structured CBN Grinding Wheel

**DOI:** 10.3390/ma17235985

**Published:** 2024-12-06

**Authors:** Zixuan Wang, Zhenshuai Li, Yang Zhao, Ji Zhao, Jiahui Du, Tianbiao Yu, Jun Zhao

**Affiliations:** 1 School of Mechanical Engineering and Automation, Northeastern University, Shenyang 110819, China; wangzx@mail.neu.edu.cn (Z.W.);; 2 Liaoning Provincial Key Laboratory of High-End Equipment Intelligent Design and Manufacturing Technology, Shenyang 110819, China; 3 School of Machinery, Shenyang Institute of Engineering, Shenyang 110136, China; 4 College of Mechanical Engineering, Zhejiang University of Technology, Hangzhou 310023, China

**Keywords:** structured grinding wheel, 3D printing, CBN, supersonic vibration, surface roughness

## Abstract

The abrasives of traditional grinding wheels are usually randomly arranged on the substrate, reducing the number of effective abrasive grains involved in the machining during the grinding process. However, there are some problems such as uneven distribution of chip storage space, high grinding temperature, and easy surface burn. In trying to address this issue, an ultrasonic vibration 3D printing method is introduced to fabricate the structured CBN (Cubic Boron Nitride) grinding wheel. The effects of the fabricated process parameters, overlap rate, scanning path, and ultrasonic amplitude were analyzed. The effects of laser power, scanning speed, and powder disk rotation speed on the topography of the printing layer were analyzed by orthogonal tests. The obtained data were input into the GA-BP (Genetic Algorithm-Back Propagation) neural network for training, and the trained model was utilized to derive the optimal process parameters. Then, the experiments were carried out to optimize the overlap rate and the scanning path. The effect of ultrasonic vibration amplitude on the surface topography and the microhardness of the printing layer was observed and investigated. The structured CBN grinding wheels were fabricated using the optimal parameters, and the performance of the grinding wheels was evaluated. The workpiece surface roughness ground by the grinding wheel fabricated with ultrasonic vibration was smaller than that without ultrasonic vibration, and a better workpiece surface quality was obtained.

## 1. Introduction

With the development of society, the manufacturing industry has higher and higher requirements for the surface quality of mechanical parts and machining accuracy [1,2]. Although more precision machining methods have been proposed [3], the grinding technology is still a particularly important method with both high surface quality and a high material removal rate [4,5]. As a tool for grinding, most of the world’s industrial powers have invested many resources in research on grinding wheels [6,7]. The traditional grinding wheel, affected by old manufacturing methods, low abrasive grain strength, and uncontrollable abrasive grain arrangement, results in shorter service life and poorer grinding performance [8]. It does not meet the high quality and precision requirements for mechanical parts in the manufacturing industry, which makes the demand for high-performance grinding wheels increasingly urgent [9,10].

The abrasives of conventional grinding wheels are usually randomly arranged on the substrate, resulting in a reduction in the number of effective abrasive grains actually involved in the machining during the grinding process. Problems appear such as the uneven distribution of chip storage space, high grinding temperature, and easy burning of the surface [11,12,13]. In order to solve these problems, many scholars at home and abroad have carried out a series of studies on grinding wheels. The researchers [14] manufactured the initial structured grinding wheel by grooving to make grinding chips easier to remove. Later, electroplating [15,16], brazing [17], and other methods were used to manufacture grinding wheels. This type of method can be designed according to the needs of the abrasive grain arrangement. However, these methods have the disadvantages of complex processes and environmental hazards, etc. 3D printing technology as an emerging manufacturing technology has been utilized in many industries and fields [18]. Compared with other manufacturing technologies, 3D printing technology can save resources and achieve personalized design in structural design. Therefore, 3D printing technology can be used to manufacture structured grinding wheels.

In this paper, the current research status of structured grinding wheels will be presented in terms of structuring in the manufacturing process of grinding wheels (direct method) and structuring in the surface finishing of grinding wheels (indirect method) [19].

Rabiey [20] machined grooves on the surface of conventional grinding wheels. It was found that less abrasive grains on the surface of the structured grinding wheels were involved in the scratching and ploughing action during the grinding process, which reduced the grinding temperature and avoided workpiece burns. In addition, in the grinding experiment on the 100Cr6 workpiece using the structured grinding wheel with a contact area of 25%, the grinding force was reduced by more than one-third. This indicates that the surface structured grinding wheel can effectively reduce the grinding force. Walter et al. [21] processed different groove structures on the surface of a conventional metal–ceramic bond CBN grinding wheel using laser ablation. The grinding wheels with different groove types were applied to grind 100Cr6 material. It was found that the grinding force decreased significantly. In addition, the grinding force stability of grinding wheels with a grooved structure on the surface is also better than that of conventional unstructured grinding wheels. The average value of the radial profile of the grinding wheel had little change, indicating that the wear rate of the groove structured grinding wheel did not increase significantly.

Tsuchiya et al. [22] designed and fabricated a grinding tool with a spiral groove structure. The spiral grooves on the surface of the grinding wheel can discharge the chips generated during the grinding process in time, effectively avoiding the clogging of the grinding wheel. The grinding quality of the workpiece surface was greatly improved. The surface roughness of the workpiece reached Ra 32 nm. Oliveira et al. [23,24] designed the surface structure of the grinding wheel according to the surface texture of the workpiece, and then processed the specific texture pattern onto the grooved grinding wheel. By changing different relative speeds between the workpiece and the structured grinding wheel during the grinding process, different specific texture patterns could be machined on the workpiece surface.

Song et al. [25] investigated the effect of grooved grinding wheels on grinding temperature. The experiments proved that grinding wheels with a spiral groove structure can effectively reduce thermal damage. At the same time, the selection of reasonable groove parameters according to the processing requirements of the workpiece can improve the grinding performance of the grinding wheel, and reduce the fluctuation of the grinding force during the grinding process. Wu et al. [26] used pulsed laser heating technology and ultrasonic vibration impact technology to simulate the thermal and mechanical impacts generated during the grinding process. The research results show that the grinding force shows periodic fluctuation due to the intermittent contact between the grooved grinding wheel and the workpiece surface. It is favorable to the workpiece surface to produce brittle micro-fracture removal.

Traditional fine-grained diamond grinding wheels are prone to clogging when grinding optical glass and other materials. Therefore, Zhao et al. [27,28,29] carried out the innovative dressing of grinding wheels, which resulted in nano-scale surface roughness after grinding microcrystalline glass and optical glass with precision-dressed large-grained diamond grinding wheels. Guo et al. [30,31,32] utilized a picosecond laser for the microstructuring of large-grained diamond grinding wheels. Multiple parallel grooves were machined on the surface of the workpiece, which increased the number of cutting edges per unit area of the grinding wheel and facilitated the realization of plastic grinding. Compared with the traditional unstructured grinding wheels, the microstructured grinding wheels can significantly reduce the subsurface damage of the optical glass workpiece.

Aurich et al. [15,16] manufactured structured CBN electroplated grinding wheels, and carried out the kinematic simulation of grinding wheels with different morphologies or numbers and arrangement orders of abrasive grains on the surface to study the effect of different parameters on the grinding performance. The grinding test showed that the grinding wheel with an ordered arrangement of abrasive grains can greatly reduce the grinding temperature and grinding force. Heinzel et al. [33] studied the plated structured diamond grinding wheel with an ordered arrangement of abrasive grains, and used the finishing adjusted plated diamond grinding wheel to grind the optical glass BK7. The surface roughness of the workpiece was obtained at less than Sa 20 nm. Burkhard et al. [17] fabricated a single-layer honing tool with an ordered arrangement of abrasive grains, which was used for grinding the 16MnCr5 material at a grinding speed of 1–3 m/s, a feed rate of 3 m/min and a depth of 0.4 mm. The experimental results show that although the concentration of abrasive grains of the grinding tool is reduced by 25% compared with that of ordinary grinding wheels, it can still maintain good grinding ability. The grinding efficiency and tool life were improved, and the machining time was reduced to less than 2/3.

Meng et al. [34] prepared a single-layer brazed diamond grinding wheel with an orderly arrangement of abrasive grains for grinding and polishing, and carried out comparative experiments on Q345 steel grinding with a traditional resin grinding wheel. The results show that although the noise of the prepared grinding wheel increases compared with the traditional resin grinding wheel, the shape of its grinding chips is regular and the grinding efficiency is greatly improved. Deng et al. [35] designed a new structured diamond grinding wheel, and it was prepared by combining the powder injection molding technology and vacuum brazing technology. The surface arrangement of diamond fibers was optimized by performing a finite element simulation of the grinding process. The experimental results show that the diamond fiber grinding wheel reduces the surface roughness of the workpiece surface by 20% and the residual stress on the surface by 8~12% compared with the ordinary resin grinding wheel.

Lyu et al. [36,37] combined the bionic leaf sequence theory with the grinding mechanism to prepare a sunflower disk seed arrangement structured grinding wheel. The results showed that the grinding performance of the leaf sequence arrangement grinding wheel was better than that of the traditional grinding wheel. The grinding performance of the grinding wheel can be improved by changing the leaf sequence coefficient. Yuan et al. [38] prepared a regular arrangement electroplated grinding wheel with abrasive grain groups by using a selective composite electrodeposition process method. The composite mask was used to solve the problem of the abrasive group showing a mushroom-type arrangement, and the multilayer abrasive grains could not be produced. Grinding comparison experiments were carried out using the prepared grinding wheels and the conventional electroplated grinding wheels. The results show that the grinding temperature of the abrasive grain controllable arrangement grinding wheel is significantly lower than that of the conventional electroplated grinding wheel. The gaps between the abrasive grain clusters can accommodate a large amount of abrasive chips and become a channel for abrasive chips to be discharged. The phenomenon of grinding wheel clogging is avoided, and good grinding performance of the grinding wheel is maintained.

Yang et al. [39,40] prepared diamond grinding wheels with an ordered arrangement of abrasive grains using selective laser sintering technology. The results of the grinding test showed that the grinding force of the wheel was uniform, and there was no shedding of diamond grains. However, the diamond grinding wheels sintered with a lower power laser are prone to grain shedding, and too high a laser power can cause the graphitization of diamond grains. Qiu [41] used three-dimensional light-curing technology to prepare diamond grinding wheels. Its abrasive grains were arranged in concentric circles, helixes, and rectangles, which realized the three-dimensional controllable arrangement of the diamond abrasive grains and enhanced the life of the grinding wheel. Tian et al. [42] prepared grinding wheels with octahedral through holes by using laser selective zone melting technology. While ensuring the mechanical strength of the grinding wheel, the through hole also increases the chip space. It reduces the grinding temperature, and improves the grinding quality. Zhang et al. [43] prepared a ceramic bonded porous diamond grinding wheel by using slurry direct-write molding technology, which has a controllable and uniform distribution of porosity, and effectively improves the chip removal ability of the grinding wheel. Wang et al. [44] introduced laser cladding remelting technology to fabricate a no-impact trajectory structured CBN grinding wheel. It can increase the fluid pressure at the grinding wheel outlet. The chemical metallurgical reactions among the CBN grain, metal bond, and substrate were formed, and the micro-mechanism was analyzed using element distribution measurement and XRD analysis.

With the development of ultrasonic vibration technology in various processing technologies, such as grinding [45] and polishing [46,47], the using of 3D printing methods and ultrasonic vibration assistance technology provides new ideas for the fabrication of structured grinding wheels. In this paper, orthogonal experiments with the GA-BP neural network method were used to obtain the optimal 3D printing process parameters of the CBN grinding wheel. The round-trip scanning path was selected, which has a better surface quality. The effect of varying ultrasonic vibration powers on microhardness and surface topography of the printing layer was examined. With the fabricated CBN grinding wheel, the grinding surface analysis was conducted.

## 2. The Fabrication Method and Experiments

### 2.1. The Experimental Equipment

The 3D printing system for the structured CBN grinding wheel mainly consists of a KUKA robot, fiber laser, powder feeder, and ultrasonic vibration system. The ultrasonic vibration system consists of an ultrasonic generator, a transducer, and a variable amplitude rod. The printing trajectory is firstly drawn by PQart software (V10, Beijing C.H.L Robotics Co., Ltd., Beijing, China), and then the optimized trajectory program is imported into the control system of the KUKA robot, which moves according to the preset trajectory with its equipped laser cladding head and powder feeder nozzle. At the same time, the grinding wheel substrate is connected to an ultrasonic vibration system with a variable amplitude rod. The ultrasonic vibration system and the KUKA robot work together to complete the 3D printing. The specific parameters of ultrasonic-vibration-assisted structured CBN grinding wheel 3D printing system are shown in Table 1.

The BRANSON 2000bdc ultrasonic generator (BRANSON Ultrasonics Corporation, Danbury, CT, USA) was used in this study. The operating voltage is 200~240 V. The amplitude fluctuation is less than or equal to ±2%. The output frequency is 20 kHz, and the maximum ultrasonic amplitude A_0_ is 15 μm. The power ratio r of the ultrasonic generator is adjustable from 0% to 100%. The power ratio and the amplitude have a linear relationship, so the amplitude can be adjusted indirectly by adjusting the power ratio.

The grinding test is conducted on a DMG DMU50 5-axis machining center (Seebach, Thuringia, Germany) with a Siemens 840DSL operating system. The maximum spindle speed is up to 18,000 rpm, which meets the requirements of the experiment.

### 2.2. The Characterization Equipment

The microstructures of the printing layer and the surface of the ground surface were examined by laser confocal microscopy (LEXT OLS4000, Olympus Corporation, Tokyo, Japan). The microhardness at the cross-section of printing workpiece was measured using a Vickers indentation tester (MH-500, Shanghai Hengyi Precision Instrument Co., Ltd., Shanghai, China) with seven measurement points from the surface of the substrate to the surface of the printing layer.

### 2.3. The Fabrication Materials

#### 2.3.1. The Substrate

The substrate is the main part of the grinding wheel, and the bonding agent is firmly bonded to the substrate surface by chemical metallurgical bonding under the action of a high-energy laser. First of all, the melting point of the substrate material should be able to close to that of the bonding agent in order to form a good chemical metallurgical bond. Secondly, the substrate material should have enough strength to ensure that it will not be damaged and cracked under the impact of grinding force during the grinding process. Therefore, AISI 1045 steel is selected as the grinding wheel substrate material. Its yield strength and tensile strength are high, and it is easy to form a melt pool because of its small laser reflectivity.

#### 2.3.2. The Abrasive Grains

**Figure 1:**
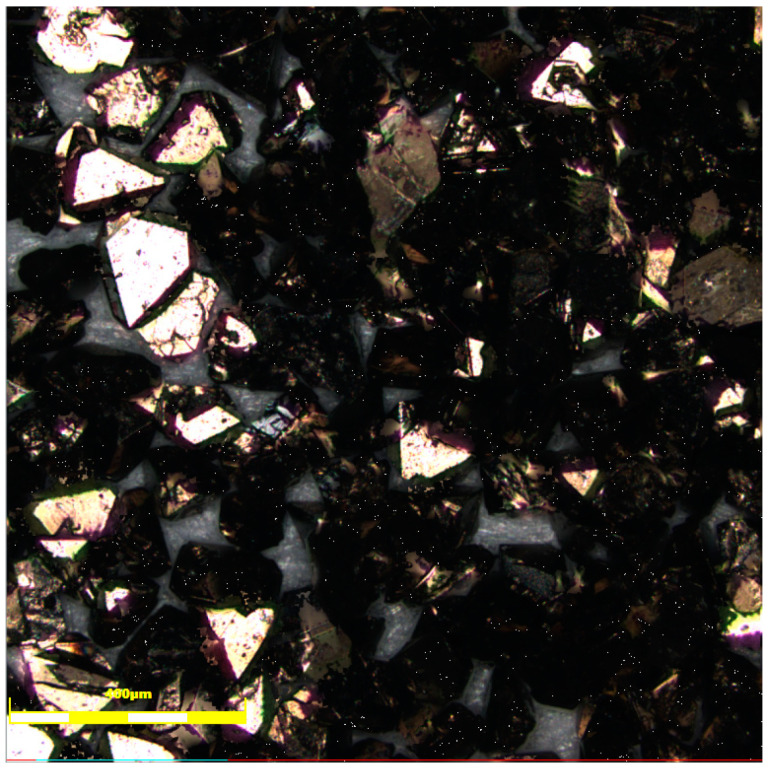
The topography of CBN-980T abrasive grains.

The CBN abrasive grains have good chemical stability. Compared with diamond, it is less likely to react with iron group elements, and has stable performance at high temperatures, so the CBN abrasive grains are more suitable [48]. In this study, CBN-980T titanium-plated abrasive grains from Funek Superhard Materials Ltd. (Zhengzhou, China). were selected. Titanium is able to react chemically with CBN as well as the bonding agent to improve the bonding agent’s hold ability with CBN abrasive grains. To ensure the flow of abrasive grains during the printing process, suitable abrasive grain size should be taken. The grain size with 100 to 120 mesh was selected, and the abrasive grain morphology can be seen in Figure 1.

#### 2.3.3. The Metal Bond

The Cu-Sn-Ti metal bond contains a large amount of titanium, which is easy to react with CBN abrasive grains and forms a strong bond. Therefore, The Cu-Sn19-Ti10 alloy powder is selected as the metal bonding agent with a particle size of −100~+250.

### 2.4. The Fabrication Method

**Figure 2:**
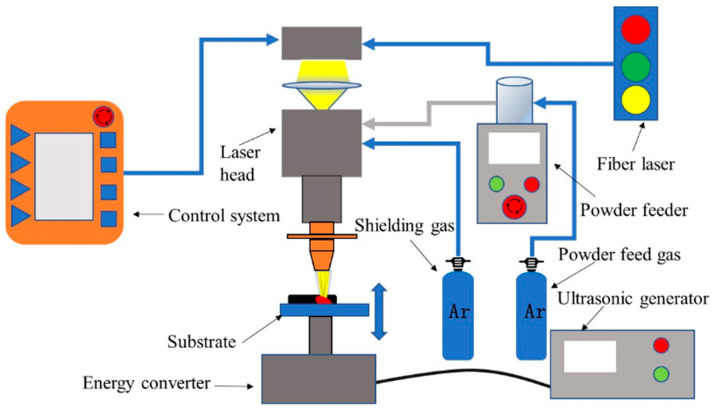
Ultrasonic-vibration-assisted 3D printing schematics of structured grinding wheels.

During the 3D printing process, the chemical metallurgical bonding occurs between the powder, abrasive grains, and the substrate under the action of high-energy laser. This is a sudden heating and cooling process. The flow rate of molten metal in the melt pool is large, and the addition of ultrasonic vibration changes the printing layer topography and the abrasive grain distribution. Figure 2 shows the schematic of an ultrasonic-vibration-assisted structured grinding wheel 3D printing system. The 3D printing system of the structured CBN grinding wheel is mainly composed of a robot arm, fiber laser, powder feeder, and ultrasonic vibration system. The robot arm moves with the laser head and the powder feeder nozzle according to the preset trajectory. The grinding wheel substrate is connected with the ultrasonic vibration system through the thread and conducts vertical ultrasonic vibration.

Because of the high melting point of the substrate and the high temperatures that tend to cause CBN abrasive grain burns, a secondary printing method is used. First, a layer of bonding agent is printed on the substrate, called the bonding agent layer. Then, the mixture of the CBN abrasive grain and bonding agent is printed on the bonding agent layer, called the abrasive layer. This allows the abrasive layer and the bonding agent layer to form a melt pool at a lower temperature while preventing CBN abrasive burns. Ultrasonic propagation in the metal melt pool will cause many complex nonlinear effects, mainly including thermal effects, acoustic flow enhancement effects, and cavitation effects.

## 3. The Printing Process Analysis

### 3.1. The Process Parameter Optimization

#### 3.1.1. The Experimental Design

In the 3D printing process of the ultrasonic-assisted structured CBN grinding wheel, factors such as laser power, powder feeding speed, and scan speed affect the quality of the printing layer. These factors affect each other, which together determine the dimensions of the printing layer such as height, width, and depth. Therefore, in this study, the process parameters suitable for grinding wheel printing were selected through orthogonal experiments and the GA-BP neural network model. According to the previous studies, the laser power was selected in the range of 180~340 W, the scan speed was selected in the range of 2~6 mm/s, and the powder disk speed was selected in the range of 0.8~2.0 r/min. A 3-factor and 5-level experimental scheme were selected, as shown in Table 2. The powder carrier gas flow rate of the powder feeder was adjusted to 4 L/min to ensure the optimal printing effect. The protective gas flow rate was adjusted to 15 L/min to prevent metal powder as well as CBN abrasive grains from burning and deteriorating in the high-temperature environment during the printing process.

#### 3.1.2. The Microtopography Analysis

**Figure 3:**
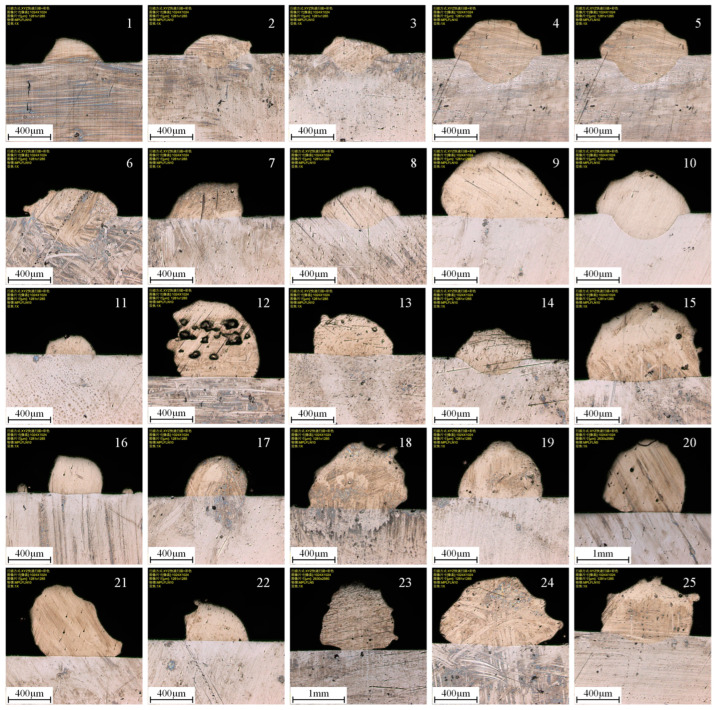
The cross-sectional topography of the printing layer (Scanning mode: XYZ fast scanning + color; Image size [pixels]: 1024 × 1024; Image size [um]: 1281 × 1285 for 1–19, 21–22, 24–25 and 2630 × 2580 for 20, 23; Objective: MPLFLN10 for 1–19, 21–22, 24–25 and MPLFLN5 for 20, 23; Zoom: 1×).

In order to ensure a good bonding effect between the printing layer and the substrate, it is necessary to ensure that the wetting angle between the printing layer and the substrate is in the range of 0° < θ < 90°. The shape coefficient of the printing layer was in the range of η > 2. The dilution rate γ should be less than 50%. Because the moving speed of the robot arm changes greatly at the beginning and end of printing, the middle part of the single printing layer was selected for observation. The cut workpiece was ground and polished, and the cross-sectional topography of the printing layer is shown in Figure 3. Then, the melt width, height, and depth of the printing layer were observed and measured. The orthogonal experiment results are shown in Table 3.

#### 3.1.3. The Optimization Results Based on GA-BP Neural Network Method

(1) GA-BP neural network principle

Each of the neural network algorithms and genetic algorithms has its own advantages. Using the neural network algorithm can quickly find the optimal solution, but the neural network selects a wide range of initial values and often finds a local optimal solution, while the genetic algorithm has a stronger global search ability. Therefore, the advantages of the genetic algorithm can be used to make up for the shortcomings of the BP neural network. The two methods can be used in combination. Firstly, the genetic algorithm is used to select all the initial values in a large range to find the optimal value, and then the BP network is used to find the exact solution in a small range [49]. This method can significantly reduce the computational error of the neural network and has greatly improved the training speed, convergence of the network, and the final optimization search results [50]. In this study, a three-layer BP neural network was used to solve the problem. There is a certain approximate relationship between the number of nodes n2 in the implicit layer and the number of nodes n1 in the input layer [51].

(1) $$n_{2}=n_{1}\times 2+1$$

**Figure 4:**
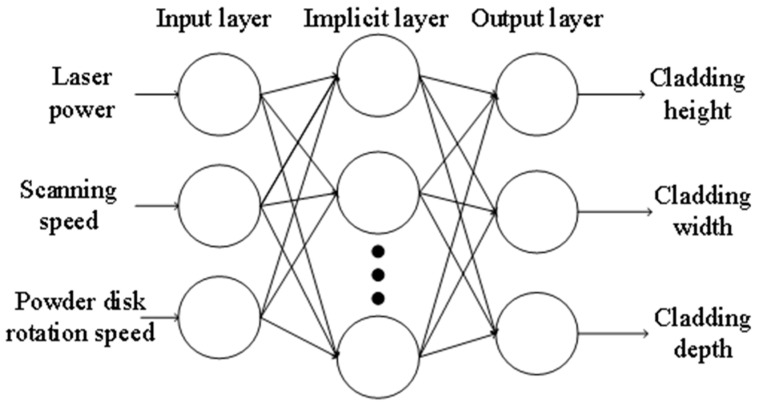
The BP neural network structure.

According to the above equation, the number of nodes in the input layer is determined to be 3 and the number of nodes in the implicit layer is determined to be 7, so the structure of this neural network is 3 × 7 × 3. This neural network has three output ports and its BP neural network structure is shown in Figure 4. The GA-BP prediction algorithm parameters can be seen in Table 4.

(2) GA-BP neural network model training and simulation

Due to the large variation in the collected data, they should be normalized according to Equation (2) in order to eliminate the effect of singular data on the model.

(2) $$x=\frac{x-x_{\min}}{x_{\max}-x_{\min}}$$

where *x*_max_ is the maximum value of the sample; *x*_min_ is the minimum value of the sample.

**Figure 5:**
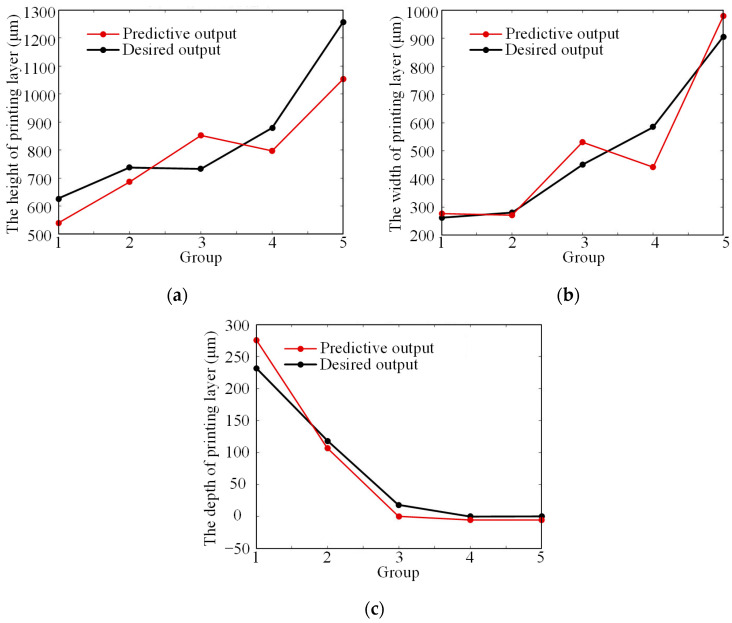
The comparison of predictive and desired outcomes: (**a**) the height of printing layer; (**b**) the width of printing layer; (**c**) the depth of printing layer.

Based on the relevant results obtained from 25 groups of experiments, groups 2, 8, 13, 18, and 23 were selected as test data, and the remaining 20 groups were used as training data. The data were trained using the GA-BP neural network, in which the first 20 groups were loaded as inputs and outputs. The training was completed when the training results met the required accuracy or the maximum number of iterations was reached. The neural network was tested using the remaining 5 groups of test data. The curves of predicted values and actual values for the height, width, and depth of the printing layer were obtained, as shown in Figure 5. The prediction model established using the BP neural network can accurately describe the effects of different process parameters on the width, height, and depth of the printing layer, so as to predict the printing results. After the parameter optimization by the model, the final process parameters for a single-pass printing layer are determined as follows: the laser power P is 300 W; the scanning speed vs. is 4.5 mm/s; and the powder disc rotational speed is 1.5 r/min.

### 3.2. The Effect of Overlap Rate

**Figure 6:**
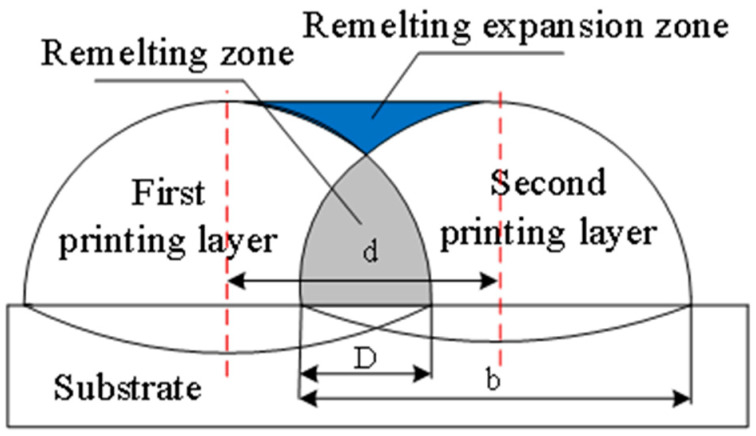
The overlap schematic diagram of printing layer.

The overlap schematic diagram between the two printing layers is shown in Figure 6, where the remelting will occur at a high temperature when printing the second layer. The remelting has a significant effect on the height and surface flatness in this zone. The overlap ratio can be expressed by Equation (3):

(3) f=D/b=(b−d)/b

where *D* is the overlap width. *b* is the width of the printing layer, and *d* is the distance between the centers of the two single-pass printing layers.

**Figure 7:**
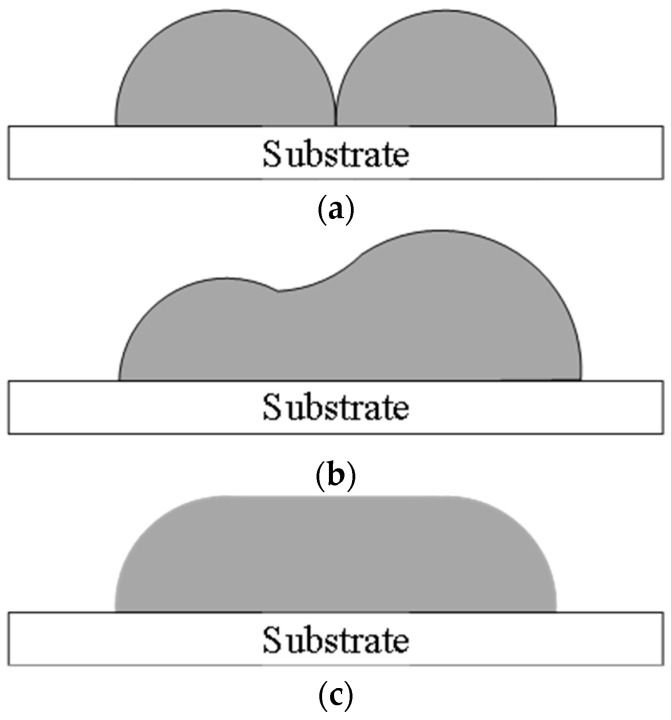
The effect of different overlap ratios on the printing layer: (**a**) a small overlap ratio; (**b**) a large overlap ratio; (**c**) a moderate overlap ratio.

The remelting expansion causes an increase in the height of the expansion zone, and the best results are obtained if the increased height of the expansion zone is exactly equal to the height of the single-pass printed layer. If its increased height is less or greater than the height of the single-pass printing layer, then it will have an effect on the surface profile of the printing workpiece, thereby increasing its roughness. By adjusting the overlap rate, different surface profiles of the 3D printing layer can be obtained, as shown in Figure 7.

**Figure 8:**
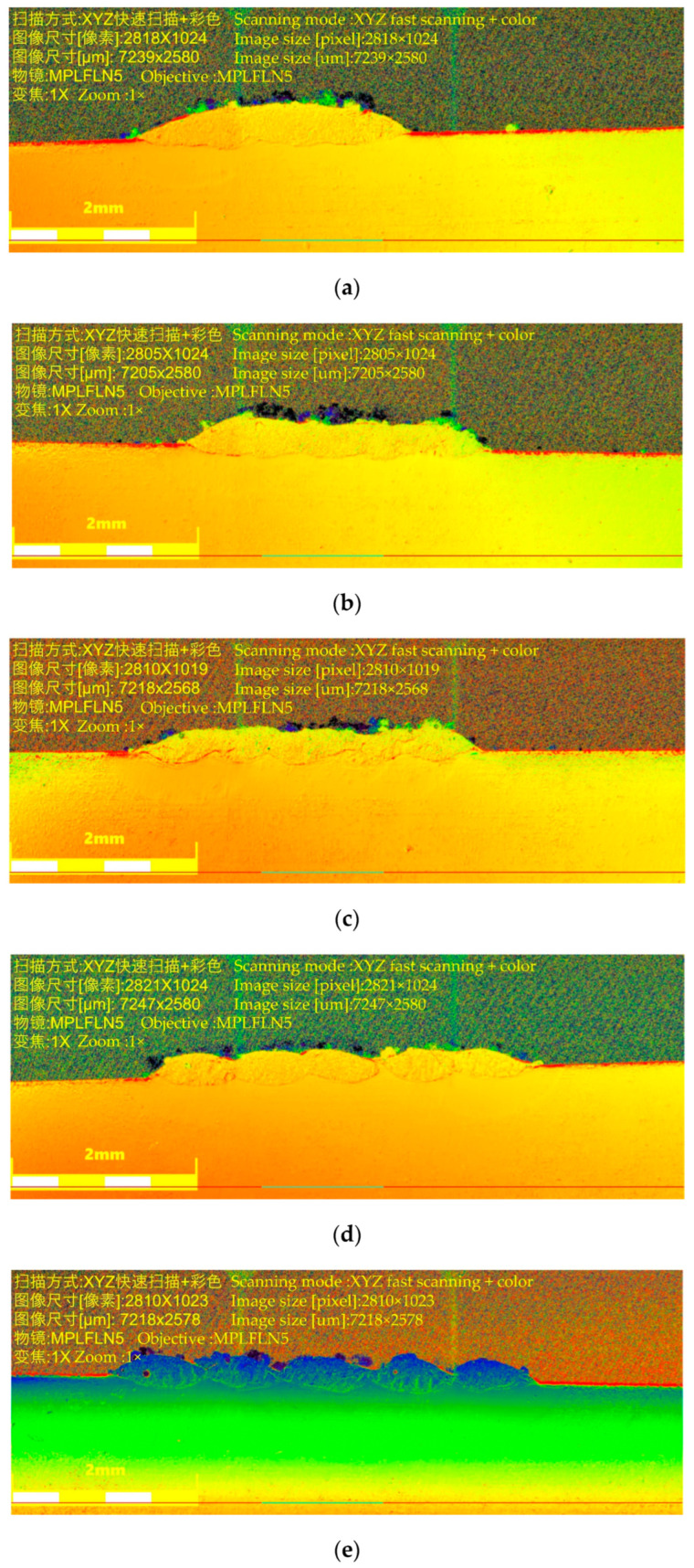
The cross-sectional topography of the printing layer with different center distances: (**a**) 0.5 mm; (**b**) 0.6 mm; (**c**) 0.7 mm; (**d**) 0.8 mm; (**e**) 0.9 mm.

According to the optimization results of the process parameters above, the experimental process parameters of the overlap rate are laser power of 300 W, laser scanning speed of 4.5 mm/s, powder disk rotation speed of 1.5 r/min, powder feed gas flow rate of 4.0 L/h, and protective gas flow rate of 20 L/h, as shown in Table 5. The printing workpiece was processed by wire cutting, sandpaper grinding, polishing, and cleaning, and the cross-section topography of the printing layer under different overlap rates was observed under the microscope, as shown in Figure 8.

As can be seen in Figure 8a,b, the overlap ratio is too large, and the printing layer increases in height due to remelting expansion. As shown in Figure 8c, the surface of the printing workpiece is relatively flat and the remelting expansion height is not much different from the height of the printing layer. However, the overlap ratio is too small, as shown in Figure 8d,e, where the surface of the printing layers appears to be concave. Therefore, a printing center distance of 0.7 mm and an overlap rate of 31.2% were selected.

### 3.3. The Effect of Scanning Path

**Figure 9:**
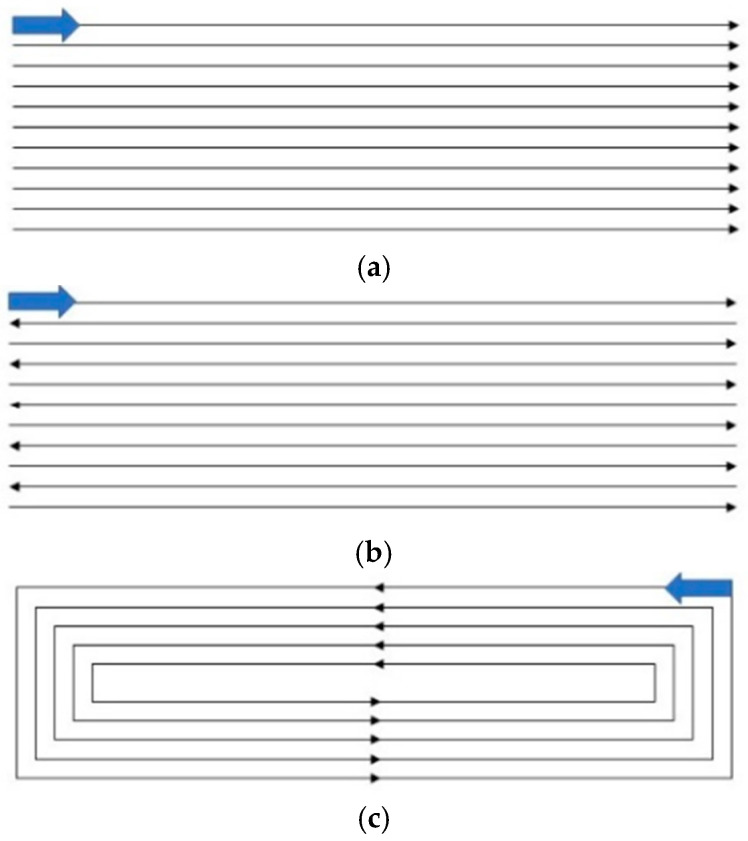
The schematic diagram of different scanning paths: (**a**) unidirectional scanning; (**b**) round-trip scanning; (**c**) scanning from the outside in.

Different scanning methods have different effects on the quality of the printing layer. Adopting different scanning methods during the printing process will result in different heating and cooling times of the substrate; thus, it affects the thermal deformation of the substrate and the amount of powder on the surface of the printing layer. Using the optimal overlap ratio of 31.2% (center distance d = 0.7mm) obtained above, laser power of 300 W, scanning speed of 4.5 mm/s, and powder disk rotation speed of 1.5 r/min, a layer of rectangle (8 × 30 mm) was printed along different scanning paths, as shown in Figure 9.

**Figure 10:**
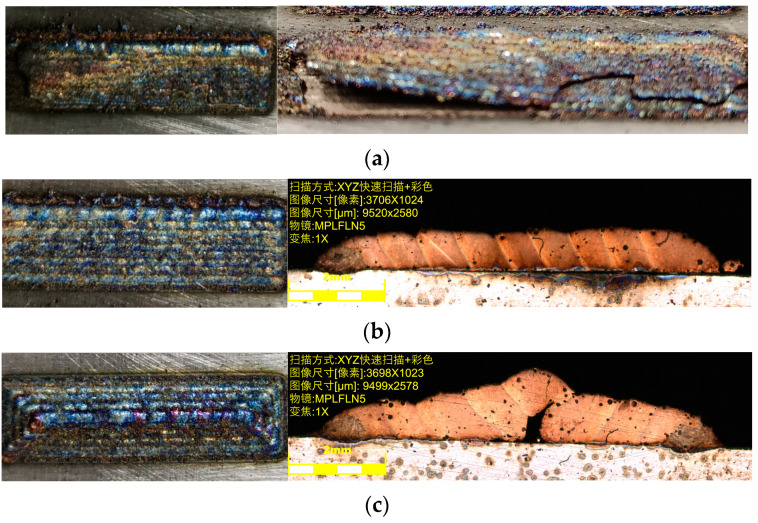
The cross-sectional topography of the printing layer with different scanning paths: (**a**) unidirectional scanning; (**b**) round-trip scanning (Scanning mode: XYZ fast scanning + color; Image size [pixels]: 3706 × 1024; Image size [um]: 9520 × 2580; Objective: MPLFLN5; Zoom: 1×); (**c**) scanning from the outside in (Scanning mode: XYZ fast scanning + color; Image size [pixels]: 3698 × 1023; Image size [um]: 9499 × 2578; Objective: MPLFLN5; Zoom: 1×).

As shown in Figure 10a–c, the cross-sectional morphologies of the printing layer by three different scanning methods were obtained with the size of 8 mm × 30 mm. It can be seen in Figure 10a that the whole printing layer is upwardly warped from the printing start direction. Due to the better heat dissipation conditions at the two end positions of the printing layer, there is no preheating and slow cooling process. This results in a high cooling rate and high stress values at both ends. Moreover, for the unidirectional scanning, the time between the two printing layers and the cooling time is long, resulting in a large stress value at both ends of each printing layer. This ultimately leads to the cracking of the printing layer due to excessive stress.

As shown in Figure 10b, the round-trip scanning yields the best surface quality of the printing layer, which has less sticky powder on the surface and no obvious overburning phenomenon. The round-trip scanning method causes more heat to accumulate in the printing layer, and the flow rate of the melt pool becomes larger. More heat can be transferred to the substrate, which increases the temperature of the substrate near the melt pool. The temperature difference is reduced, and the temperature gradient is lowered, thus relieving the stress concentration phenomenon. From Figure 10c, due to the blockage of the outer printing layer, the internal printing area is heavily powdered. As a result, the remelting expansion in the inner printing area accumulates continuously, which ultimately makes the printing layer present a surface topography with uniform flatness all around and convexity in the center. There is an obvious gap in the center of the overlap zone, which is caused by the accumulation of remelting expansion in the innermost region. In summary, the round-trip scanning path was selected as the laser printing scanning method for the fabrication of grinding wheel.

### 3.4. The Effect of Ultrasonic Vibration

#### 3.4.1. The Experiment Design with Different Ultrasonic Amplitude

Based on the experimental results above, a single-factor experiment on ultrasonic amplitude was conducted. The power ratios of the ultrasonic generator were selected from 0% to 50% as shown in Table 6, and corresponding ultrasonic vibration amplitudes can be calculated by Equation (4). In order to facilitate the observation of CBN abrasive grain distribution under a microscope, each process parameter was used to print a layer of five-pass planes with the overlap ratio and scanning path determined above.

(4) $$A=A_{0}\times r$$

where *A* is the ultrasonic amplitude, *A*_0_ is the maximum ultrasonic amplitude, and *r* is the power ratio.

#### 3.4.2. The Result Analysis

**Figure 11:**
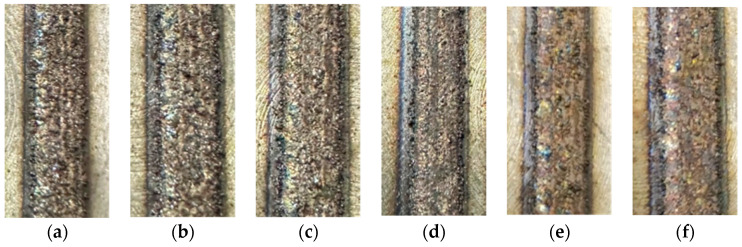
The surface topography of the printing layer with different power ratios: (**a**) r = 0%; (**b**) r = 10%; (**c**) r = 20%; (**d**) r = 30%; (**e**) r = 40%; (**f**) r = 50%.

It can be seen in Figure 11a that the surface of the printing layer is rougher with more sticky powder on the surface when the power ratio is zero. This is due to the fact that the larger molten metal droplets adsorb the smaller droplets, resulting in larger droplets, and causing the uneven surface. As the ultrasonic amplitude increases, the surface of the printing layer gradually becomes smooth. As shown in Figure 11d, when the power ratio r = 30%, it can be seen that the smoothness of the surface of the printing layer has been significantly improved. This is because the ultrasonic vibration makes the larger molten metal droplets rupture under the high-frequency vibration, so that other droplets cannot be adsorbed, and the surface of the printing layer becomes smooth.

The performance of a grinding wheel is affected by a number of factors, among which the height of the abrasive grains is one of the most important factors. The difference in material properties between the bond of the grinding wheel and the CBN grains results in different wear rates during the grinding process. If the wear rate of the bond is too fast, then it will lead to a high edge height of the grains. The holding ability of the bond for the CBN grains is reduced, resulting in the premature falling off of the CBN grains. If the wear rate of the bond is less than that of the CBN grains, then it will lead to a low edge height of the grains, and a reduction in the grinding efficiency.

The CBN grains as super-hard abrasives wear slowly in grinding. At the same time, the structured grinding wheel has grooves on the surface of it, which has enough space to hold chips, so there is no need to worry about grinding wheel clogging and self-sharpening. Therefore, enhancing the hardness of the bond helps to reduce its wear rate in the grinding process and increases the service life of the grinding wheel. Ultrasonic vibration refines the microstructure of the printing layer and enhances the hardness. Therefore, the hardness of the printing layer is used as an indicator of the print quality of the grinding wheel. A distance of 100 μm from the top of the printing layer was used as the first measurement point, followed by measurements at 100 μm intervals. A total of seven locations, numbered 1 to 7, were taken from the top of the print layer to the substrate for hardness measurements.

**Figure 12:**
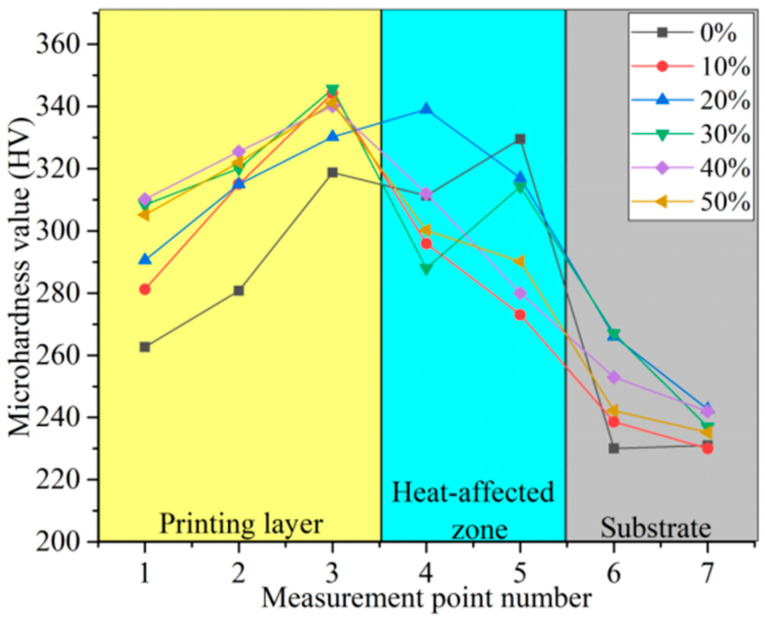
The microhardness values with different ultrasonic amplitudes (power ratios).

Figure 12 shows the microhardness values of the printing layer at different ultrasonic amplitudes. The measurement points cover the print layer, heat-affected zone, and substrate. The trend of the microhardness values with positions at different amplitudes is the same, and only the magnitude of the change is different. In the printing layer zone, the hardness increases with depth. In the heat-affected zone, the hardness value decreases compared to the printing layer zone but is higher than the substrate zone. This is because the heat-affected zone is repeatedly heated and cooled during the printing process, which is equivalent to multiple heat treatments. Therefore, its hardness is higher than that of the substrate zone. The hardness value of the substrate zone is around 240 HV.

When the power ratio was increased from 0% to 10%, the hardness at each point within the printing layer zone increased significantly. The maximum microhardness value reached 344.2 HV, which is because the ultrasonic vibration induced the dendrites in the melt pool to fracture and generate equiaxial crystals, which had the effect of fine crystal strengthening. With the increase in power ratio in the range of 10% to 50%, the increase in microhardness in the printing layer zone was not significant. This is because the amplitude of ultrasonic vibration was sufficient to fracture the dendritic crystals at a power ratio of 10%. The increasing amplitude could no longer refine the crystalline grains.

## 4. The Fabrication of Structured CBN Grinding Wheel

**Figure 13:**
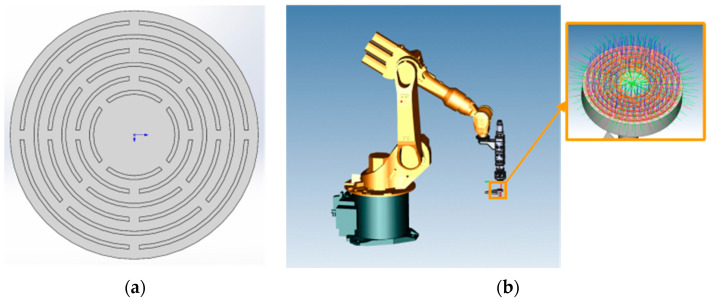
The design of structured grinding wheel: (**a**) designed structure; (**b**) PQ art trajectory programming.

Based on the selected process parameters, the overlap rate, the scanning path, and the ultrasonic amplitude, the CBN abrasive end-face structured grinding wheels were fabricated. The structured layer is six interrupted concentric rings, as shown in Figure 13a. The rings are divided into four segments to prevent the printing layer from being too long to generate stress concentration at both ends of the printing layer, which affects the performance of the grinding wheel. The six interrupted concentric rings are staggered in intermittent positions to prevent the accumulation of printing errors at both ends of the printing layer. The grinding wheel substrate is made of 45 steel. The diameter of the grinding wheel is 50 mm, and the thickness is 10 mm. The overall length of the holder is 40 mm with thread in the top half. The trajectory is programmed as shown in Figure 13b.

**Figure 14:**
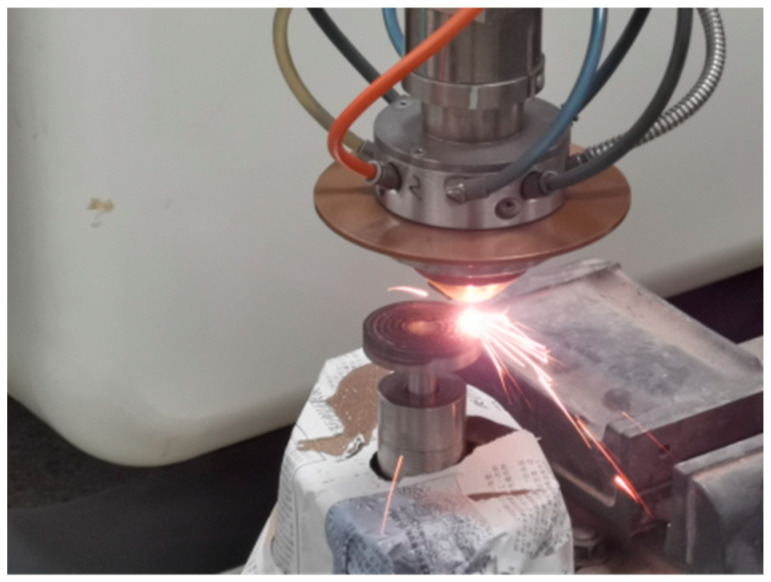
The fabrication process of the CBN grinding wheel.

The trajectory program was imported into the KUKA robot and the relevant parameters were adjusted to the optimal parameters. The grinding wheel fabrication was carried out as shown in Figure 14. In the fabrication process of the grinding wheel, the temperature of the substrate rose rapidly. In order to prevent the high temperature from damaging the machining precision, the grinding wheel was cooled down sufficiently for a period of time after each printing.

**Figure 15:**
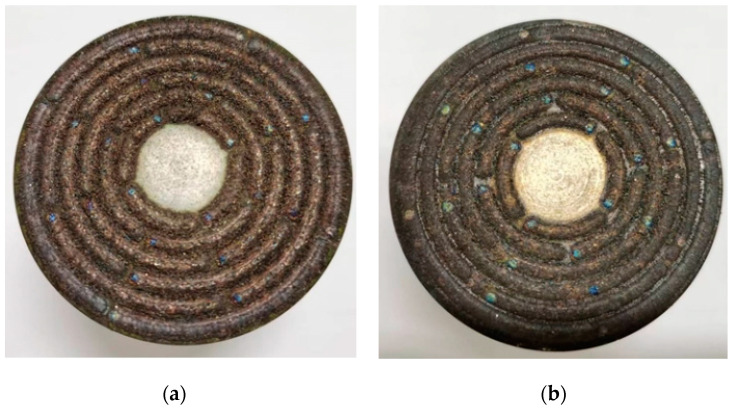
The 3D printed structured CBN grinding wheel: (**a**) without ultrasonic vibration; (**b**) with ultrasonic vibration.

The fabricated grinding wheels are shown in Figure 15. It can be seen that after applying ultrasonic vibration, the phenomenon of sticking powder between the two rings is significantly improved, and the surface of the printing layer is smoother.

## 5. Grinding Experiments Analysis

### 5.1. The Experimental Design and Setup

**Figure 16:**
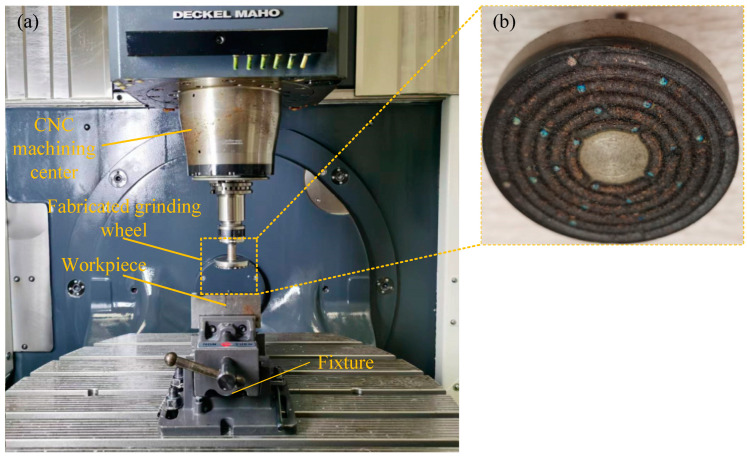
The grinding process with fabricated CBN grinding wheel: (**a**) the grinding process using a CNC machining center; (**b**) the fabricated structured CBN grinding wheel.

The grinding process with the fabricated CBN grinding wheel can be seen in Figure 16. The grinding experiments were carried out with and without ultrasonic-assisted fabrication of grinding wheel, respectively. The rotational speed of the grinding wheel was 3000 r/min, the feed speed was 40 mm/min, and the depth of cut was 30 μm. In order to avoid the unevenness of the workpiece surface to be ground, its surface was milled before the grinding was carried out to make its surface flat.

### 5.2. The Surface Quality

**Figure 17:**
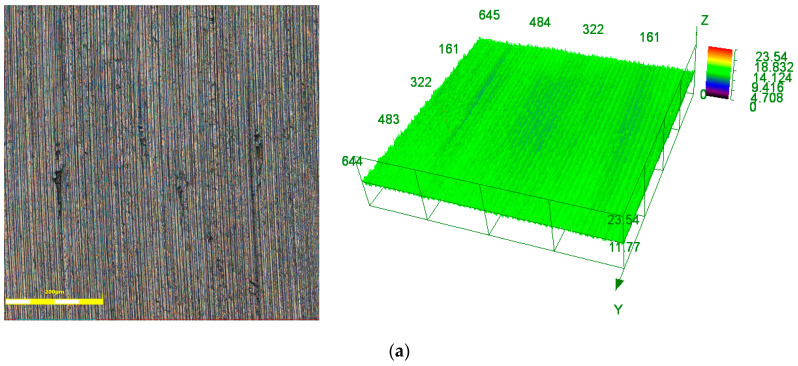

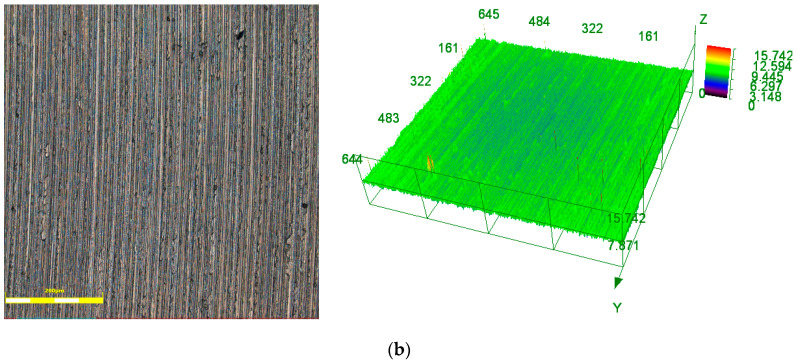
The microstructure of the ground surface (**a**) without ultrasonic vibration; (**b**) with ultrasonic vibration.

After the grinding experiment, the surface of the workpiece was observed and analyzed by laser confocal microscopy. Because both ends of the workpiece were only ground by the outer part of the end face of grinding wheel, the middle part of the ground workpiece was selected for microstructure observation and analysis. Figure 17 shows the microstructure of the surface of the ground workpiece by the grinding wheel fabricated without and with ultrasonic vibration. The ground surface by the grinding wheel without ultrasonic vibration had defects, which were caused by the uneven surface of the grinding wheel and the different protruding heights of the abrasive grains.

The measurement results showed that the line roughness of the surface ground by the grinding wheel fabricated with ultrasonic vibration (Ra 0.294 μm) was smaller than that without ultrasonic vibration (Ra 0.453 μm). In order to further analyze the surface quality of the workpiece, the 3D arithmetic mean deviation method was used as the evaluation index to conduct analysis. The surface roughness of the surface ground by the grinding wheel fabricated with ultrasonic vibration (Sa 0.303 μm) was also smaller than that without ultrasonic vibration (Sa 0.458 μm), which agrees with the results of the line roughness analysis. The reason is the uneven distribution of CBN abrasive grains on the surface of the grinding wheel fabricated without ultrasonic vibration, and the difference in the protruding height of the abrasive grains is large. For the abrasive grains with high protrusion height, the bond has poor holding power, and the CBN abrasive grains easily fall off during the grinding process. This causes defects on the surface of the workpiece, and at the same time makes the surface roughness value of the workpiece larger. The surface of grinding wheel fabricated by ultrasonic vibration is smooth. The height of the abrasive grain protrusion is more uniform and the CBN abrasive grains are uniformly distributed. The bond has stronger holding force to CBN abrasive grains. Therefore, the surface quality of the ground workpiece is better.

In this paper, the ultrasonic-vibration-assisted 3D printing method of a structured CBN grinding wheel and its grinding performance were studied. The method can achieve solid chemical metallurgical bonding among the CBN grain, metal bond, and substrate under the action of a high-energy laser. Moreover, this method can be used for complex structure design and automatic fabrication, which saves the raw materials of the metal bond and abrasive grains and reduces the cost. However, due to the high temperature and the 3D printing process, there are certain requirements for the type and grain size of the abrasive grains. In follow-up research, the motion law and distribution of CBN grains on the surface of the printing layer can be further studied by means of experimental or numerical simulation.

## 6. Conclusions

In this study, structured CBN grinding wheels are fabricated by a 3D printing method with ultrasonic vibration technology. The fabrication process was analyzed, and a grinding experiment analysis was conducted with the fabricated structured CBN grinding wheel. The main conclusions are summarized as follows:

(1) The orthogonal experiments with the GA-BP neural network method were used to conduct the parameter optimization in the 3D printing process of the CBN grinding wheel. The best combination of process parameters for the single-pass printing layer was laser power of 300 W, scanning speed of 4.5 mm/s, and powder disk rotation speed of 1.5 r/min. The overlap ratios experiments were conducted. When the center distance b = 0.7 mm, the surface of the printing layer was relatively flat, and the height of the remelting expansion was not much different from the height of the printing layer.
(2) For equal-area single-layer printing, different scanning paths had different effects on the quality of printing layer. The whole printing layer of unidirectional scanning was upwardly warped from the printing start direction due to a large stress value at both ends of each printing layer. The internal printing area was heavily powdered for scanning from the outside in. The remelting expansion in the inner printing area accumulated continuously, which ultimately made the printing layer present a surface topography with convexity in the center. The round-trip scanning path was selected as the laser printing scanning method for the fabrication of the grinding wheel, which yielded the best surface quality. It had less sticky powder on the surface and no obvious overburning phenomenon.
(3) The microhardness and surface topography of the printing layer were examined and assessed, considering varying ultrasonic vibration power ratios. When the power ratio was zero, the printing layer’s surface was rougher and had more sticky powder on it because the larger molten metal droplets absorbed the smaller ones, creating an uneven surface. The printing layer’s surface smoothed out as the ultrasonic amplitude rose. The printing layer’s surface smoothness greatly increased when the power ratio was r = 30%. The microhardness values exhibited a consistent trend at varying amplitudes from the printing layer to the substrate. The hardness increased dramatically at every location within the printing layer zone when the power ratio was raised from 0% to 10%. The highest microhardness value measured was 344.2 HV. The printing layer zone’s microhardness did not significantly increase as the power ratio increased between 10% and 50%.
(4) Ultrasonic vibration was used in the fabrication of the structured CBN grinding wheels. The results of the grinding experiments indicated that the workpiece surface ground by the ultrasonic-vibration-fabricated grinding wheel had a lower line roughness (Ra 0.294 μm) and face roughness (Sa 0.303 μm) than the workpiece surface ground by the traditional grinding wheel (Ra 0.453 μm and Sa 0.458 μm). The primary cause of this was that the ultrasonic-vibration-fabricated grinding wheel had a smoother surface and a more uniform abrasive grain protrusion height.

## Figures and Tables

**Table 1:** The specific parameters of experimental equipment.

Instrumentation	Model
Robot Control System	KUKA ZH30/60 III (KUKA Robotics Corporation, Ltd., Augsburg, Germany)
KUKA robot	KUKA-KR16-3 (KUKA Robotics Corporation, Ltd., Augsburg, Germany)
Laser device	RC52 (Nanjing Zhongke Raycham Laser Technology Co., Ltd., Nanjing, China)
Laser generator	IPG YLR-500-WC (IRE-Polus, Fryazino, Russia)
Powder feeder	RC-PGF-S (Nanjing Zhongke Raycham Laser Technology Co., Ltd., Nanjing, China)
Laser head water cooling system	MCWL-25DTR-05-1225 (Sanhe City Tongfei Refrigeration Equipment Co., Ltd., Sanhe, China)
Ultrasonic generating equipment	2000bdc (BRANSON Ultrasonics Corporation, Danbury, CT, USA, USA)

**Table 2:** The orthogonal experiment scheme.

No.	Powder Disc Speed r/min	Laser Power W	Scan Speed mm/s
1	0.8	180	2
2	0.8	220	4
3	0.8	260	6
4	0.8	300	3
5	0.8	340	5
6	1.1	180	6
7	1.1	220	3
8	1.1	260	5
9	1.1	300	2
10	1.1	340	4
11	1.4	180	5
12	1.4	220	2
13	1.4	260	4
14	1.4	300	6
15	1.4	340	3
16	1.7	180	4
17	1.7	220	6
18	1.7	260	3
19	1.7	300	5
20	1.7	340	2
21	2	180	3
22	2	220	5
23	2	260	2
24	2	300	4
25	2	340	6

**Table 3:** The orthogonal experiment results.

No.	The Width of Melt Pool µm	The Height of Melt Pool µm	The Depth of Melt Pool µm
1	589.71	239.12	13.40
2	629.68	194.29	129.54
3	627.49	163.02	180.90
4	825.68	359.72	248.05
5	818.78	276.93	288.08
6	471.77	154.11	0.00
7	714.20	341.86	13.40
8	738.94	285.88	118.38
9	1094.88	612.10	0.00
10	878.84	401.98	247.93
11	478.40	180.89	0.00
12	703.16	714.64	0.00
13	732.08	381.90	22.33
14	734.34	279.23	156.39
15	1057.07	721.49	37.96
16	509.52	355.11	0.00
17	571.84	386.40	0.00
18	878.94	614.20	0.00
19	785.40	524.82	0.00
20	1489.83	1376.14	0.00
21	761.13	627.59	0.00
22	611.95	375.18	0.00
23	1256.86	1255.16	0.00
24	1032.44	649.87	0.00
25	844.20	516.45	91.67

**Table 4:** The GA-BP prediction algorithm parameters.

Parameters	Values
Population size	40
Maximum number of iterations	80
The probability crossover	0.71
The probability of mutation	0.01
Number of trainings	1000
Training objectives	0.01
Learning rate	0.1

**Table 5:** The overlap rate experimental results.

No.	Power W	Scanning Speed mm/s	Powder Disk Rotation Speed r/min	Center Distance mm	Horizontal Overlap Rate %
1	300	4.5	1.5	0.5	50.8
2				0.6	41.0
3				0.7	31.2
4				0.8	21.3
5				0.9	11.5

**Table 6:** The printing parameters with different power ratios.

No.	Power W	Scanning Speed mm/s	Powder Disk Rotation Speed r/min	Power Ratio %
1	300	4.5	1.5	0
2				10
3				20
4				30
5				40
6				50

## Data Availability

The original contributions presented in this study are included in the article. Further inquiries can be directed to the corresponding authors.
